# Plant-related quality attributes affecting FcγRIIIa binding: affinity chromatography analysis of rituximab glycovariants from *Nicotiana benthamiana*

**DOI:** 10.3389/fpls.2025.1607403

**Published:** 2025-06-27

**Authors:** Sara Tengattini, Aurora Tini, Carla Marusic, Francesca Rinaldi, Isabella Senini, Enrica Calleri, Virginia Perez, Claudio Pisano, Marcello Donini, Caterina Temporini

**Affiliations:** ^1^ Laboratory of Pharmaceutical Analysis, Department of Drug Sciences, University of Pavia, Pavia, Italy; ^2^ Division of Biotechnology, ENEA Casaccia Research Center, Rome, Italy; ^3^ Special Product’s Line Spa, Anagni, Italy

**Keywords:** plantibodies, monoclonal antibodies, rituximab, glycosylation, glycoengineering, FCγRIIIa receptor, antibody-dependent cell cytotoxicity, affinity chromatography

## Abstract

Monoclonal antibodies (mAb) produced in plants, known as plantibodies, represent a cost-effective alternative to conventional mammalian cell cultures. Glycoengineering processes are needed to alter N-glycosylation, avoiding plant-typical glycans and enabling, for anti-cancer mAbs, the production of biobetters with improved antibody-dependent cell-mediated cytotoxicity (ADCC). In this study, glycovariants of the mouse/human chimeric anti-CD20 antibody rituximab were produced in *Nicotiana benthamiana* plants by transient expression using vacuum-agroinfiltration technology and LED lighting. To modify the glycosylation profile, treatment with kifunensine mannosidase I inhibitor (K) was used as well as ΔXF *N. benthamiana* plants optimized by “genome editing”. The produced plantibodies were characterized to assess their structural properties, including primary sequence and glycosylation profile. Binding to the FcγRIIIa receptor was investigated by affinity chromatography to explore plantibody ADCC. The influence of the glycosylation on FcγRIIIa receptor affinity was evaluated as well as the impact of post-translational modifications (PTMs). Both glycoengineering strategies were shown to produce mAbs with comparable or improved affinity for FcγRIIIa receptor. For the first time, different *in vivo* glycoengineering approaches have been compared through the characterization of the resulting mAbs and their affinity for FcγRIIIa receptor. This insight into the correlation among the expression system, plantibody glycoprofile, and predicted ADCC of individual glycoforms has not been previously reported and provides valuable support for the development of plant-based biosimilars.

## 1 Introduction

Monoclonal antibodies (mAbs) are generally produced in mammalian cell lines that have their own merits and setbacks. Despite the great efforts made over the past decades by many companies to scale up cell culture bioreactors, these still present several bottlenecks such as high initial investments and production costs; for this reason, alternative production methods are being explored. A promising alternative consists in the production of mAbs in plants (Shanmugaraj et al., 2020; Bharathi et al., 2024).

The use of plants to produce complex protein molecules, which may originate even from non-plant sources, is called “molecular farming” and dates back to 1989 when the first IgG1 was produced in transgenic tobacco (Hiatt et al., 1989). The production of proteins in plants has several advantages over traditional expression systems based on mammalian cells, such as low costs, ease of production, scalability, and limited risk of contamination by human pathogens (Twyman et al., 2005; Stoger et al., 2014; Shanmugaraj et al., 2020).

In the last decade, the production of antibodies in plants, known as plantibodies, has been achieved using different approaches, among which transient expression systems using both viral or *Agrobacterium*-based vectors proved to be the most successful ones (Komarova et al., 2010; Sainsbury et al., 2010; Peyret and Lomonossoff, 2013). Nevertheless, challenges such as structural differences, purification hurdles, and production consistency must be faced to enable the production of effective plant-based biosimilars.

Glycoengineering has a key role in plantibody production. The efficacy of mAbs relies partly on the ability to trigger cellular effector functions by binding, through their Fc region, to the Fcγ receptors (FcγRs) present on the surface of the immune cells. The antibody-dependent cellular cytotoxicity (ADCC) is mediated by the interaction with the FcγRIIIa receptor, mainly expressed on the surface of natural killer (NK) cells. The N-glycosylation of Asn297 is crucial for Fc binding to FcγRIIIa, and the nature of the glycan chain affects the affinity for the receptor (Polmear and Good-Jacobson, 2022).

Glycosylation in plants differs from the one in mammalian cells. Plant cells must be engineered to suppress the expression of endogenous plant-specific β1,2-linked xylose and α1,3-linked fucose, avoiding potential immunogenicity, increase in clearance, and reduction/loss of ADCC (Schähs et al., 2007; Wang et al., 2022). Besides the production of plantibodies with an N-glycosylation profile similar to the mammalian-derived mAbs, plant glycoengineering also has the potential to produce biobetters with enhanced ADCC. It is well documented that the core α1,6-fucosylation of Fc N-glycans hindered the interaction with FcγR and negatively affected the ADCC activity. Core α1,3-fucose, typical of plant glycans, seems to have a comparable effect (Schähs et al., 2007; Marusic et al., 2018). Plant cell glycoengineering and the resulting lack of fucosylation have shown the potential of producing therapeutic mAbs with enhanced ADCC compared to the mammalian-derived analogs, exposing plants as a valuable expression system not only in terms of productivity but also of product quality (Schähs et al., 2007; Marusic et al., 2018).

Recent research studies showed that terminal galactose improves ADCC, but its influence is significantly affected by the presence of fucose, with a higher impact on afucosylated mAbs (Zhang et al., 2020). Despite the recent advancements in plant glycoengineering, the literature shows that galactosylation is generally absent or incompletely achieved in plant-derived glyco-modified mAbs (Wang et al., 2022).

Besides Fc glycosylation, other post-translational modifications (PTMs) can also impact on plantibody activity. Methionine oxidation is shown to affect the interaction with FcRn and hence IgG half-life (Nimmerjahn et al., 2023). Only minimal effects were observed for FcγR binding, mainly involving FcγRIIa receptor (Bertolotti-Ciarlet et al., 2009). Asn325 deamidation was shown to interfere with the binding and the interaction with the effector cell through the alteration of the local three-dimensional structure of mAbs (Lu et al., 2020; Lippold et al., 2023). Additionally, oxidation of Try277 was shown to correlate with IgG Fc flexibility, which impacts FcγRIIIa receptor binding (Nimmerjahn et al., 2023). Plant-derived mAbs have demonstrated to be more susceptible to oxidation compared to mAbs expressed in CHO (Stelter et al., 2020; Tengattini et al., 2022). Methionine oxidation appears to be only marginally influenced by downstream processing strategies, suggesting that it is likely to occur *in planta* rather than during antibody extraction and storage (Stelter et al., 2020).

Given the peculiarity in plantibody PTMs (not only glycosylation) and their potential to affect ADCC, their comprehensive structural and functional characterization is of paramount importance to face the challenges posed by plant-based production and to reach an effective knowledge of the impact of the expression system on antibody structure and activity profile. The critical quality attributes (CQAs) of a (plant-derived) biosimilar could be indeed strongly affected by the production system, upstream and downstream processes.

In this work, we describe the engineering of two plant expression vectors carrying the heavy chain (HC) and light chain (LC) of the mouse/human chimeric anti-CD20 antibody rituximab (RTX), the first antibody-based drug approved for the treatment of patients with recurrent B-cell lymphomas (Reff et al., 1994), and its successful production in *N. benthamiana* plants by using a vacuum agroinfiltration system. Plant production was optimized by evaluating different LED lighting conditions in a hydroponic growth system. To achieve a modified glycosylation profile lacking typical plant sugars, we pursued two different strategies, one based on an agroinfiltration protocol that uses the kifunensine mannosidase I inhibitor (Kommineni et al., 2019), while the second was based on the use of glycoengineered *N. benthamiana* plants optimized for the glycosylation profile in which fucosyl and xylosyl transferase encoding genes were knocked-out by “genome editing” (Jansing et al., 2019). The mAb samples (including commercial Mabthera^®^ produced in mammalian cells) have been structurally characterized, with a particular focus on the glycosylation profile. A commercially available column was used to evaluate the binding to FcγRIIIa receptor by affinity chromatography. It has been demonstrated that the use of FcγRIIIa affinity chromatography is an orthogonal technique to surface plasmon resonance (SPR) for qualitative and comparative determination of the affinity for the Fc-receptor and its correlation to the ADCC-mediated effector function (Thomann et al., 2015; Bouvarel et al., 2022). Importantly, this affinity chromatography approach was recently validated as a valuable method for online monitoring of biologics/biosimilars (Narvekar et al., 2023). Here this analytical approach was used to correlate the glycoform composition of each plantibody sample with receptor affinity and to study the influence of PTMs to receptor binding and predicted ADCC. The data allowed us to compare the different expression system and, at the same time, to underline potentialities and limitations in the use of FcγRIIIa affinity chromatography for biosimilar/biobetter plantibody assessment.

## 2 Results

### 2.1 Expression of rituximab in hydroponically grown wild-type *N. benthamiana* plants using different light conditions

In order to optimize *N. benthamiana* growth, plants were cultivated using three different commercially available Model 28 LED lamps (Valoya AP673L, NS12, and G2) that have been developed to maximize the vegetative growth of different plant species. These LEDs have similar photosynthetic active radiation (PAR) characteristics but differ in the 400–700-nm light spectra, with NS12 having a reduced emission in the red spectra and an increase in the blue component while AP673L and G2 are very similar, apart from a reduced green spectrum component and an increased far-red intensity in the latter (**Supplementary Table S1**). The impact of the different lighting on growth was evaluated by analyzing the main parameters such as plant height and leaf fresh weight (**Supplementary Figure S1**). The results show that plants grown under AP673L and G2 LEDs have a faster growth rate and higher leaf mass yield (~28 g of fresh leaf weight per plant) compared to NS12 (~16 g of fresh leaf weight per plant). The lower performance of the NS12 LEDs could be attributed to the reduced emission in the red spectra (around 700 nm) which is considered the most efficient at driving photosynthesis.

The plant-codon-optimized sequences encoding for the HC and LC of rituximab and the p19 silencing suppressor (**Supplementary Figure S2A**) were cloned into the pBI-Ω plant expression vector and used to transform *Agrobacterium tumefaciens* (LBA 4404). Groups of 10 hydroponically grown wild-type *N. benthamiana* plants were cultivated under different lighting conditions (Valoya AP673L, NS12, or G2) and were co-infiltrated using vacuum with an *Agrobacterium tumefaciens* solution containing a 1:1:1 mix of bacterial clones containing the HC and LC and the p19 silencing suppressor (**Supplementary Figures S2B, C**).

**Figure 1:**
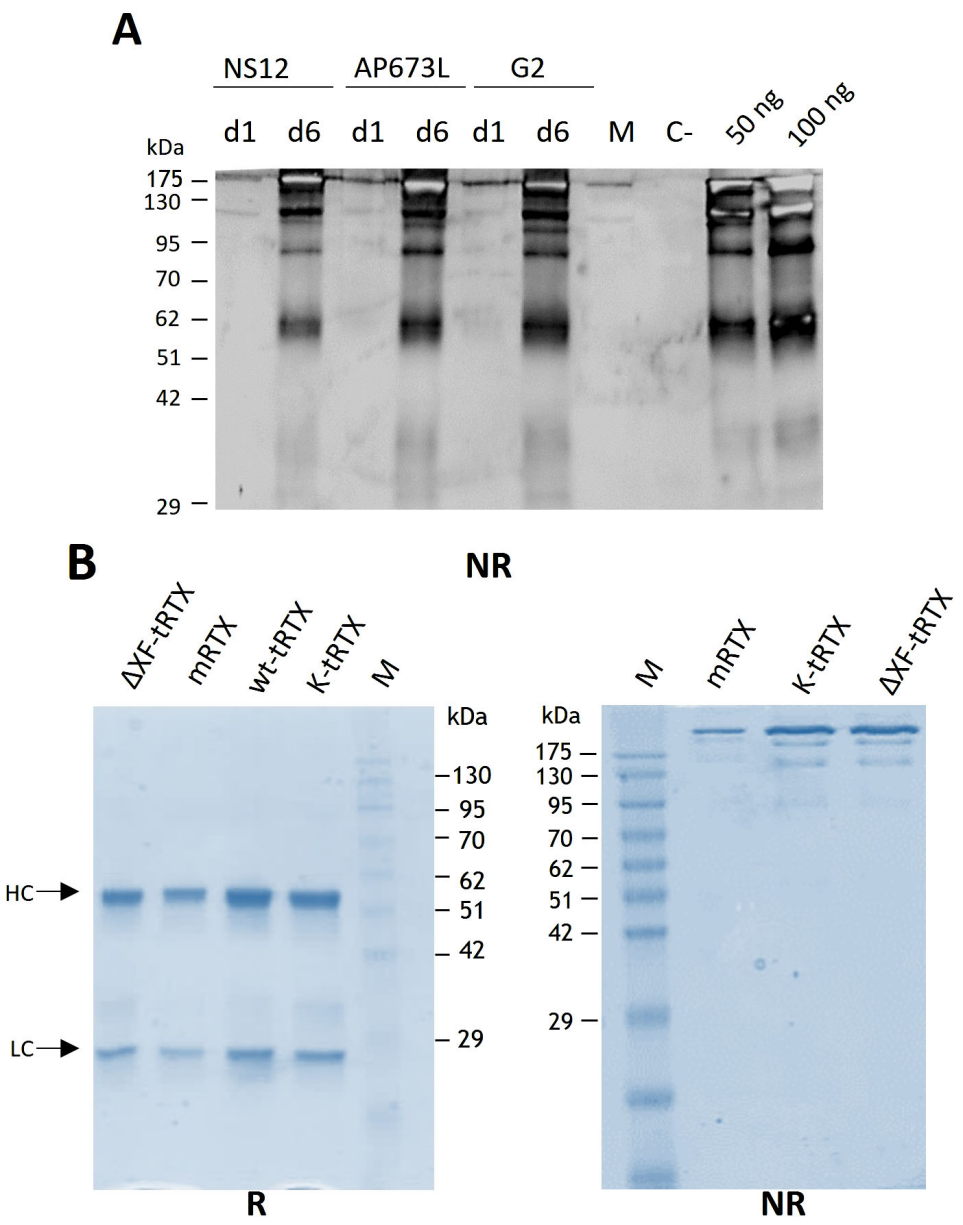
Expression and purification of rituximab in *N. benthamiana*-agroinfiltrated plants. **(A)** Western blot analysis of extracts from wild-type *N. benthamiana* plants grown under different lighting conditions (Valoya AP673L, NS12, or G2) producing rituximab (wt-tRTX). Leaves were collected at days 1 and 6 (d1 and d6) post-infiltration, and the expression of the antibody was assayed by Western blot analysis using an anti-human γ chain antibody under non-reducing (NR) conditions. C-, leaves infiltrated only with the P19 silencing inhibitor were used as a negative control. M, molecular weight marker PiNK Plus Prestained Protein Ladder (GeneDireX). **(B)** Coomassie staining of purified proteins separated by 12% SDS-PAGE under reducing (R) and non-reducing (NR) conditions. Purification was performed using a HiTrap™ FF Protein A column either from agroinfiltrated WT *N. benthamiana* leaves accumulating rituximab (wt-tRTX), leaves agroinfiltrated in the presence of the kifunensine inhibitor (K-tRTX) or from glycoengineered ΔXF plants (ΔXF-tRTX). A total of 5 µg of proteins was loaded on each lane. CHO-derived rituximab (m-RTX) was used as a control (1 µg). M, molecular weight marker.

Leaves were collected at days 1 and 6 post-infiltration (d.p.i.), and the expression of the antibody (wt-tRTX) was assayed by Western blot analysis using an anti-human γ chain antibody. Analysis under non-reducing conditions (**Figure 1A**) showed the presence on day 6 of a strong band at high molecular weight (apparently higher than 175 kDa), corresponding to the assembled immunoglobulin, and fainter bands at lower molecular mass (approx. 130, 95, and 62 kDa), most probably corresponding to minor degradation products. Antibody expression levels in the extract at day 6 post-infiltration were quantified by double-antibody sandwich (DAS) ELISA. The expression levels in plants grown under AP673L and G2 plants were comparable (94.0 ± 12 and 75.0 ± 9 mg/kg, respectively), while levels using the NS12 light were sensibly lower (37.5 ± 8 mg/kg). This result is in line with the lower growth performances observed for *N. benthamiana* plants obtained using the NS12 LEDs, highlighting the importance of light “quality”, especially the component in the red spectra, on the yield of recombinant protein. A positive impact of red light component in the accumulation of recombinant proteins was previously observed in transgenic plants expressing a recombinant antibody fragment (Zhang et al., 2023).

### 2.2 Production of rituximab glycovariants in *N. benthamiana* plants by vacuum agroinfiltration and protein A purification

Batches of 50 plants of *N. benthamiana* plants grown under the AP673L LED lights were co-infiltrated (1:1:1 mix of bacterial clones containing the HC and the LC and the p19 silencing suppressor) using a vacuum chamber. To obtain a glyco-optimized version of rituximab, batches of glycoengineered ΔXF plants were used as well as wild-type *N. benthamiana* plants agroinfiltrated in the presence of the kifunensine mannosidase I inhibitor. Leaves were collected 6 days post-infiltration (d.p.i.) and stored at -80°C.

**Figure 2:**
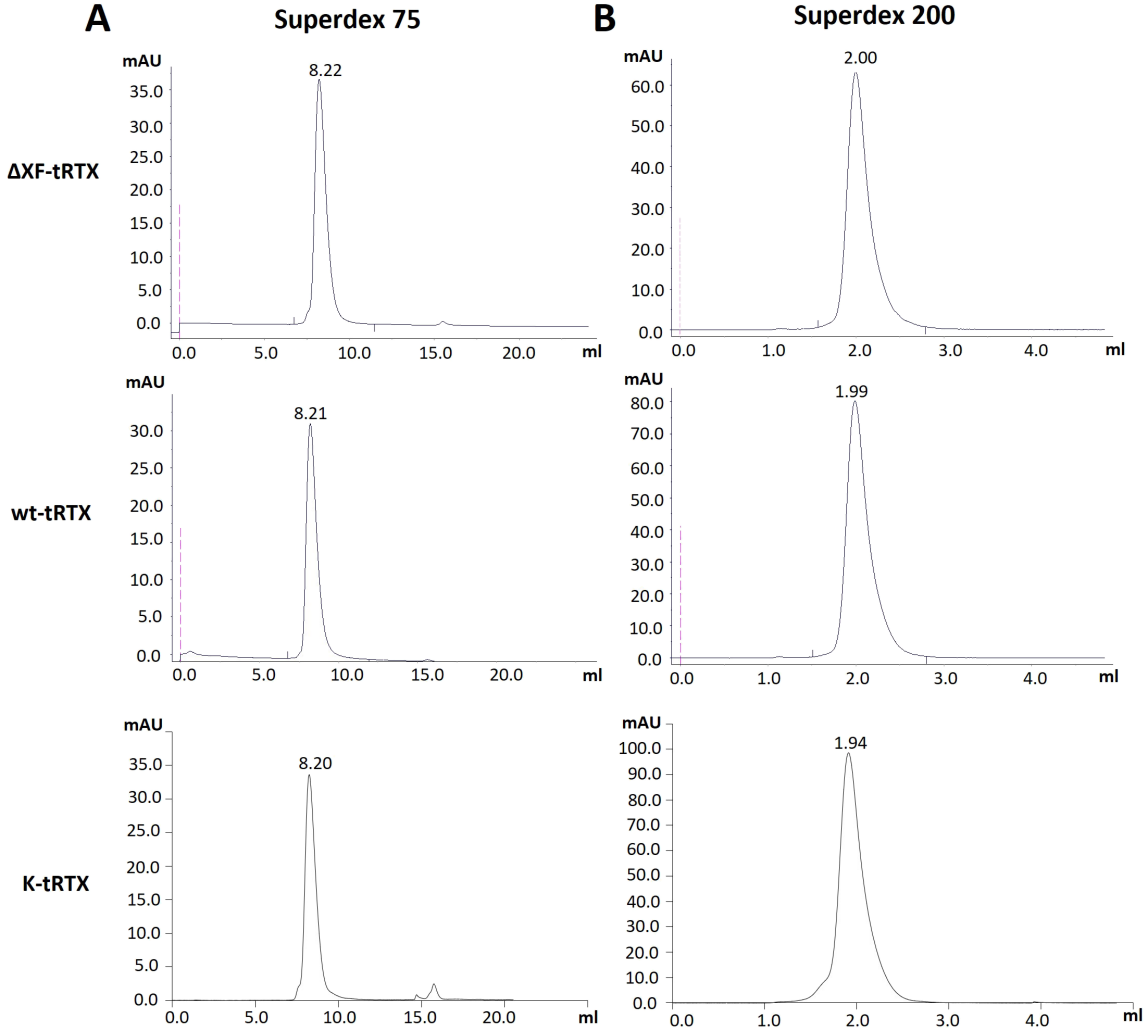
Assembly assessment of wt-tRTX-, ΔXF-tRTX-, and K-tRTX-purified antibodies by size-exclusion chromatography (SEC). Superdex™ 75 10/300 **(A)** or Superdex™ 200 5/150 GL **(B)** columns were used to assay the presence of possible aggregation and degradation products in the antibody preparations. In the SEC analysis using Superdex™ 75, all antibodies show a major peak (>98%) at about 8.2 mL as observed for the control m-RTX (**Supplementary Figure S3**).

Purification from 40-g batches of agroinfiltrated leaves was performed using a HiTrap™ FF Protein A column either from agroinfiltrated leaves accumulating rituximab (wt-tRTX) from wild-type *N. benthamiana*, leaves agroinfiltrated in the presence of the kifunensine inhibitor (K-tRTX), or ΔXF-agroinfiltrated *N. benthamiana* (ΔXF-tRTX). The average yield of final purified wt-tRTX, K-tRTX, and ΔXF-tRTX was similar, typically in the range of 2 to 3 mg, corresponding to about 50–75 mg/kg fresh weight (FW). A typical SDS-PAGE analysis of the purified antibodies under reducing (R) and non-reducing (NR) conditions is shown in **Figure 1B**. Coomassie-stained proteins in SDS-PAGE analysis under reducing conditions showed the expected bands at about 50 kDa for the HC and at about 25 kDa for the LC. These were similar to those observed for the CHO-derived RTX used as a control (m-RTX). Non-reducing SDS-PAGE analysis of the purified K-tRTX and ΔXF-tRTX antibodies revealed a major band (higher than 175 kDa) matching that of the positive control (m-RTX). All antibodies also showed bands with a slightly lower mass probably corresponding to differently glycosylated/aglycosylated products. To further assess the assembly and integrity of the purified antibodies, size exclusion chromatography (SEC) using SuperdexTM 75 10/300 or SuperdexTM 200 5/150 GL columns was performed (**Figures 2A, B**, respectively). Analysis was performed to assay the presence of possible aggregation and degradation products in the antibody preparations. The wt-tRTX, ΔXF-tRTX, and K-tRTX antibodies showed a unique peak at about 8.20 mL (**Figure 2A**), matching the result obtained for the control m-RTX (**Supplementary Figure S3**). Separation performed on SuperdexTM 200 column also showed a unique peak at about 2.0 mL for all molecules. This analysis revealed that all purified plant antibodies are in their correctly assembled form with a purity level of more than 98%.

### 2.3 Structural characterization of plantibodies

#### 2.3.1 Identity and amino acid sequence characterization

The characterization of plant mAbs has been focused on the identity and integrity assessment of the primary sequence in comparison to the commercial m-RTX. First, peptide mapping was carried out to assess the identity of the protein sequence in the tRTX tryptic digest samples. As shown in **Table 1**, a high sequence coverage (>94% in all cases) that demonstrates the consistence of the protein backbone in all plant-derived samples was obtained.

**Table 1:** Identity characterization of plant-produced RTX at both peptide level and semi-intact (reduced-IdeS-generated fragment) level.

Sample	Protein sequence coverage (%)^a^	Fragment identification^b^				Average glycosylation degree (%)^c^
		LC Da (assign.)	Fd Da (assign.)	NG-scFc Da (assign.)	G-scFc Da (assign.; %)	
m-RTX (Mabthera)	96	23035 (pLC)	25325 (pFd)	N.d.	25199 (-K; G0F; 46.5) 25361 (-K; G1F; 45.9) 25523 (-K; G2F; 7.6)	100
wt-tRTX n= 2	96	23036 (pLC)	25324 (pFd) 24008 (pQ1-T227)	23754 (-K) 23883 (+K)	25053 (-K; G0; 46.5) 25181 (G0; 20.5) 25331 (-K; G0FX; 25.7) 25459 (G0FX; 7.3)	57
K-tRTX n= 2	95	23036 (pLC)	25324 (pFd) 24008 (pQ1-T227)	23754 (-K) 23881 (+K)	25295 (-K; Man7; 5.5) 25457 (-K; Man8; 13.6) 25619 (-K; Man9; 67.9) 25749 (Man9; 12.9)	70
ΔXF-tRTX n= 5	94	23035 (pLC)	25325 (pFd)	23754 (-K) 23883 (+K)	25053 (-K; G0; 75.2) 25181 (G0; 24.8)	91

*n*, number of different preparations of the sample; N.d., not detected.

a Peptide level.

b Semi-intact (reduced-IdeS-generated fragment) level.

c Glycosylation degree is calculated as the percentage ratio between G-scFc and total scFc (NG + G) from HILIC-UV traces of reduced and IdeS-digested sample fragments.

Secondly, semi-intact analysis was also carried out on reduced and IdeS-digested mAbs that contain Fd, Fc/2, and LC fragments to get a picture of the proteoform composition of the samples including truncated species or basic/acidic variants which can be missed during peptide mapping (Camperi et al., 2021). Samples have been first analyzed by RPLC-HRMS. While the LC moiety was conserved in all samples, the Fd fragment was also revealed as truncated species. In detail, in m-RTX, the fragment mass was consistent with the expected one. For the wt-tRTX and K-tRTX samples, it was detected both as intact and truncated species with a mass of 24,008 Da, corresponding to the sequence pQ1-T227. Finally, in ΔXF-tRTX, Fd was detected as intact, with the same mass observed for the m-RTX (**Table 1**). Fc/2 fragments, which contain the glycosylation site, were observed in all of the samples with variable MWs indicating differences in the glycosylation profile, other than in the number of terminal lysine residues, as will be discussed in the next section.

Taken together, the data obtained suggest the amino acid sequence similarity of plant-based mAbs compared to m-RTX. A stability issue was highlighted by the detection of truncated Fd in the tRTX samples that might suggest higher susceptibility to proteases.

#### 2.3.2 Glycosylation profiling

To study the identity and composition of the glycoforms obtained in the different tobacco expression systems, the fragments generated by IdeS digestion and reduction were also separated in hydrophilic interaction LC (HILIC). HILIC is a chromatographic mode known for its capability to resolve glycoforms, even at intact protein level, based on the size and composition of the sugar chain (Tengattini et al., 2024). The analysis of semi-intact antibodies in HILIC-UV-MS enabled us to chromatographically separate individual glycoforms of Fc/2 fragments, which showed differences in retention times according to their glycan portion, estimate their relative abundance, and tentatively assign their identity. Glycopeptide mapping analyses were then performed to confirm glycan composition.

**Figure 3:**
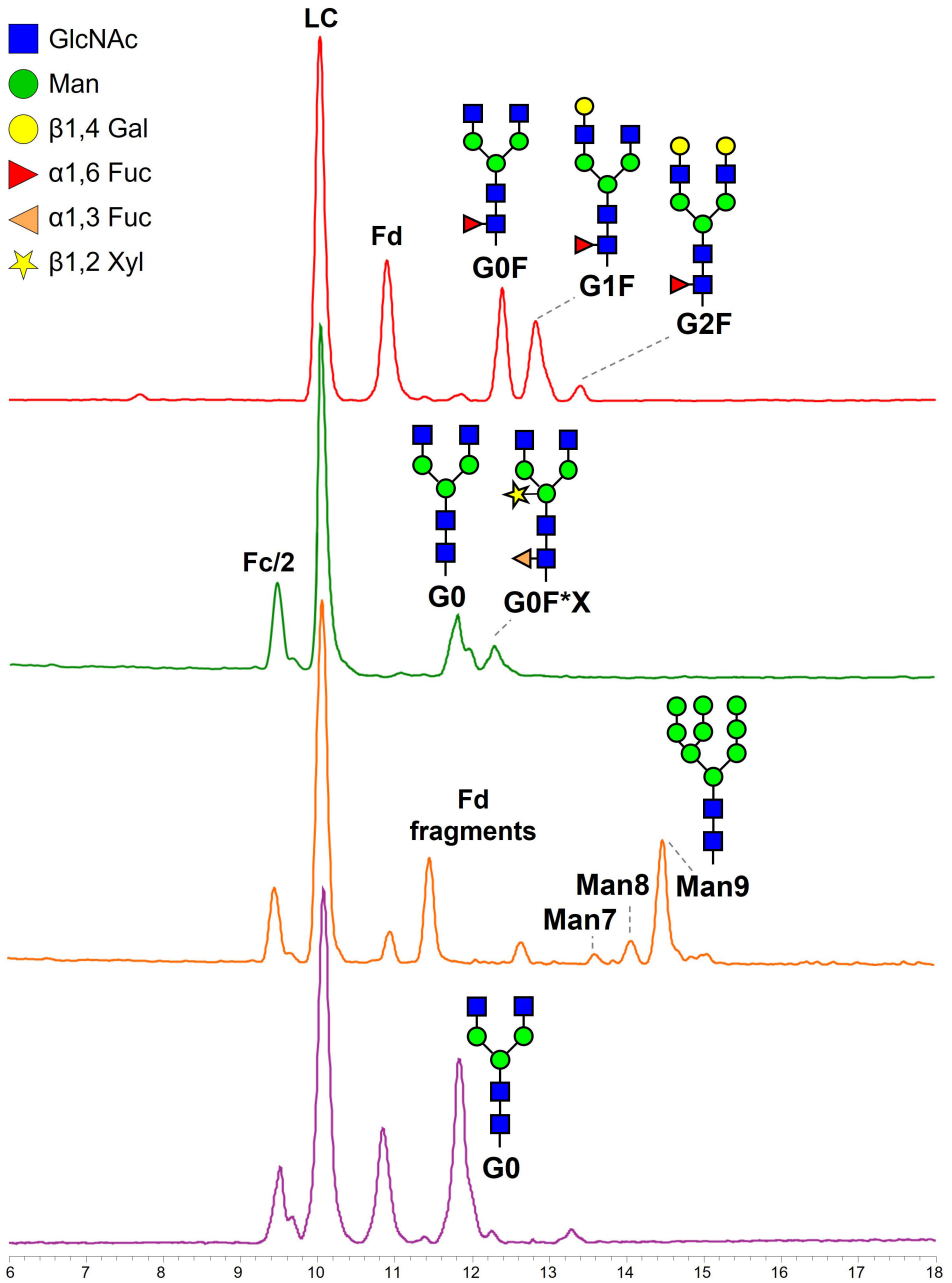
Base peak chromatograms obtained from HILIC separation of IdeS-digested and reduced m-RTX (red), wt-tRTX (green), K-tRTX (orange), and ΔXF-tRTX (purple violet). Fd, fragment d, corresponding to the heavy chain of the Fab; Fc/2, half fragment crystallizable; LC, light chain.

The glycosylation pattern of m-RTX corresponds to the expected one and shows three dominant glycoforms containing G0F, G1F, and G2F glycans (**Figure 3**; **Table 1**). The observed relative abundances also agree with the composition reported in literature (Kang et al., 2020). In the HILIC trace of wt-tRTX, besides some minor and less detectable glycoforms containing a variable number of residues of N-acetylglucosamine (GlnNAc), mannose (Man), fucose (Fuc, F), and xylose (Xyl, X), two major glycosylated species were detected, corresponding to the G0 form, and a second glycoform containing, in addition to G0, one Fuc and one Xyl (G0F*X) (**Figure 3**; **Table 1**). It should be noted that in plants fucosylation involves a core α (1-3)-linked fucose (F*), as opposed to the α(1-6)-linked fucose found in mammalian cells (F).

The addition of the α-mannosidase inhibitor kifunensine results in a K-tRTX with reduced fucosylation and xylosylation in favor of higher levels of high-mannose type N-glycans, as demonstrated by the presence of the more abundant glycoform Man9 and the less abundant Man8 and Man7 (**Figure 3**; **Table 1**). The additional peaks appearing in the chromatographic trace are related to Fd and its truncated forms. The analysis of ΔXF-tRTX shows a highly homogeneous glycosylation, characterized by the presence of the unique glycoform G0 and by the total absence of typical plant-glycans (α(1,3)F and X) (**Figure 3**; **Table 1**).

Separation of mAb fragments using HILIC also enabled the chromatographic resolution of the non-glycosylated (NG) Fc/2 fragment, which was less retained from its glycosylated counterparts. This separation allowed us to estimate the glycosylation degree as the ratio of the UV peak areas of glycosylated over the total Fc/2 fragments (**Table 1**). Non-modified tobacco plants produced 57% glycosylated mAb (number of productions (*n*) = 2). The addition of kifunensine improved glycosylation to 70% (*n* = 2), while ΔXF-tRTX samples reach a degree of 91% (*n* = 5).

The MS detection additionally revealed the coelution, under the same peak, of Fc/2 fragments differing in the presence or absence of the C-terminal lysine residue. While the C-terminal lysine was completely absent in the m-RTX, the proteoform carrying one additional lysine was consistently detected in the tRTX samples. However, it is well established that this difference is not functionally relevant.

The glycosylation pattern of tRTXs was further studied by glycopeptide mapping in HILIC-ESI-MS/MS to confirm the glycosylation site and the composition of glycan moieties already hypothesized by semi-intact MS analyses. In m-RTX the three main glycopeptides were identified consisting of peptide R.EEQYNSTYR.V bearing G0F, G1F, or G2F moieties (**Supplementary Table S2**). In wt-tRTX, glycopeptide mapping analysis confirmed the composition of the two main glycoforms, G0 and G0F*X. Some other minor glycoforms not visible at subunit level were detected thanks to the highest sensitivity of glycopeptide analysis, including G0F, Man8, and Man9 (**Supplementary Table S2**). The glycan composition of K-tRTX, with Man9 glycopeptide as major glycopeptide, followed by Man8 and Man7, and that ofΔXF-tRTX, corresponding to the afucosylated G0, were also confirmed. According to semi-intact analysis data, the non-glycosylated R.EEQYNSTYR.V peptide was detected and identified in all of the samples except for m-RTX, confirming the incomplete glycosylation of tRTX samples at a different degree.

Also, the glycopeptide analysis confirmed that glycosylation has occurred solely at the position Asn301.

### 2.4 Affinity for FcγRIIIa receptor

#### 2.4.1 Analysis of mAbs by affinity chromatography and peak assignments

Affinity chromatography on FcγRIIIa column was performed under gradient pH, resembling the physiological interaction between IgGs and the receptor (Bouvarel et al., 2022; Narvekar et al., 2023). For their identification, each eluting peak was collected, dialyzed, IdeS-digested, and reduced, and the resulting fragments were separated by HILIC-MS. Thus, a detailed picture of the glycoform ratios and their affinities was derived using the same analytical method used to characterize the whole sample.

**Figure 4:**
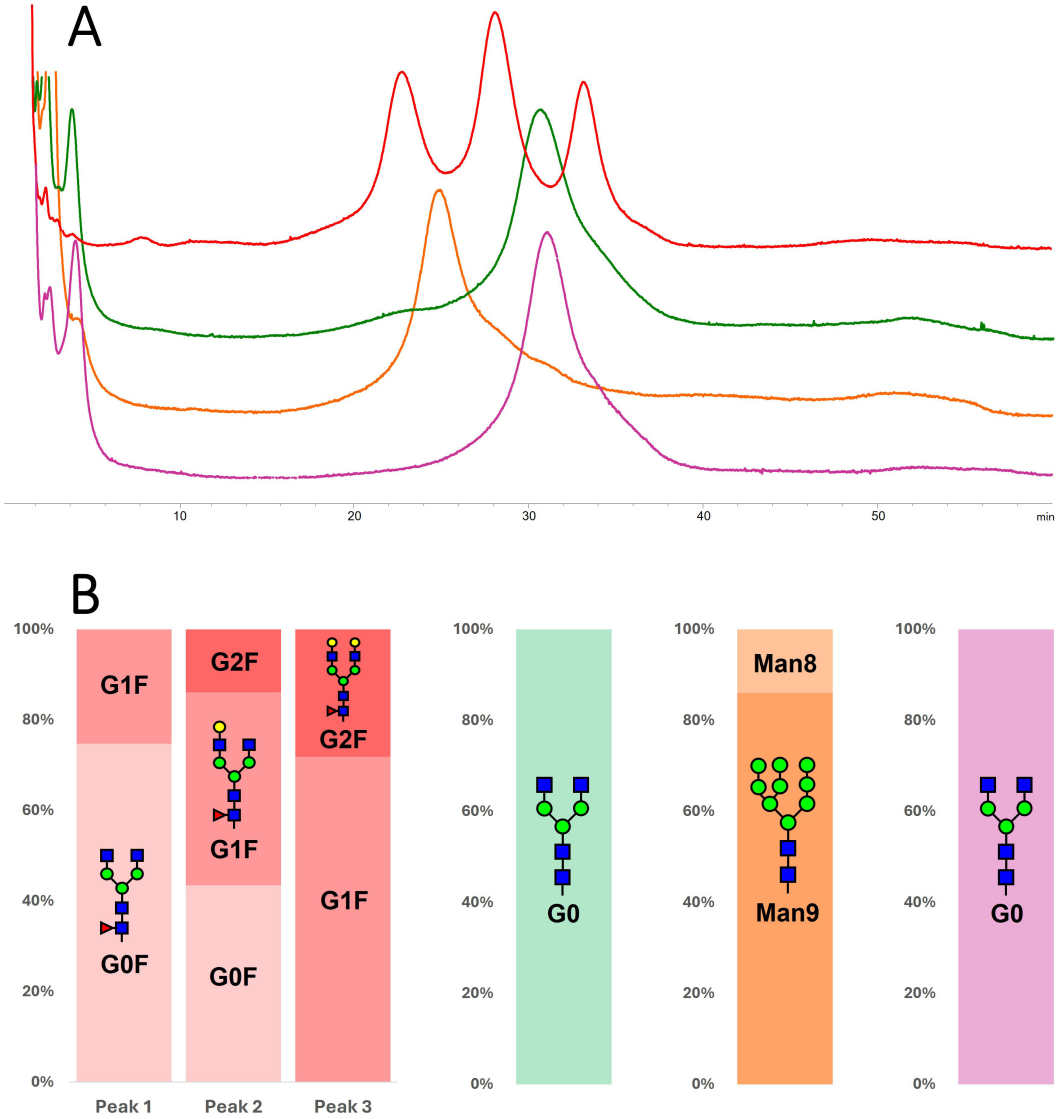
**(A)** Comparison of chromatograms obtained on the FcRIIIa column for m-RTX (red), wt-tRTX (green), K-tRTX (orange), and ΔXF-tRTX (purple violet). **(B)** Identification of glycoforms eluted under each peak; information was obtained by peak collection and middle-up HILIC separation.

The m-RTX was first considered, and under the conditions explored, three resolved peaks were detected. The first peak eluted at 22.87 min, while the others had a relative retention time (retention time normalized for the retention time of the first eluting peak; rrt) of 1.23 and 1.45, respectively (*n* = 3). An excellent repeatability was determined for both areas (mean RSD 0.5%) and rt (mean RSD 0.7%), which enables to consider any rt shift as representative for an affinity change. Identification of the eluting species (**Table 2**; **Figure 4**) reveals that the increase of galactose units (from 0 to 2) improves the affinity and thus retention time. The presence of the same glycoform under the different peaks is not ascribed to non-efficient peak focusing but rather to a highly selective retention of glycoforms bearing either the same glycans (isobaric) in diverse ramification positions (galactosylation on the 6- or 3-branch) or an asymmetric glycosylation.

**Table 2:** Chromatographic data obtained by affinity LC-UV traces on FcγRIIIa column and glycan species assignment for samples of m-RTX, wt-pRTX, K-pRTX, and ΔXF-pRTX.

Sample	Peak	RT (min) ± SD	Glycan^a^	Composition (%)^b^
m-RTX	#1	22.87 ± 0.18	G0F G1F	74 25
	#2	28.19 ± 0.21	G0F G1F G2F	43 43 14
	#3	33.22 ± 0.21	G1F G2F	72 28
wt-tRTX	#1	30.31	G0	100
K-tRTX	#1	24.95	Man8 Man9	14 86
ΔXF-tRTX	#1	30.47	G0	100

a Identity was assessed by HILIC-HRMS of IdeS-digested and reduced collected peak.

b Relative abundance of detected species under each peak.

The tRTX samples were analyzed under the same analytical conditions. wt-tRTX resulted in one single peak at rrt 1.33, whose composition was 100% G0 glycoform. This data indicates that, among the two main glycoforms in wt-tRTX, only G0 expresses affinity for the receptor, with G0F*X not being retained. The K-tRTX profile shows a major peak with rrt of 1.09 corresponding to the major Man9 and Man8 glycoforms. The ΔXF-tRTX chromatographic trace results in one single peak at rrt 1.33, corresponding to G0 (**Table 2**; **Figure 4**).

#### 2.4.2 PTMs’ influence on binding with FcγRIIIa receptor

**Figure 5:**
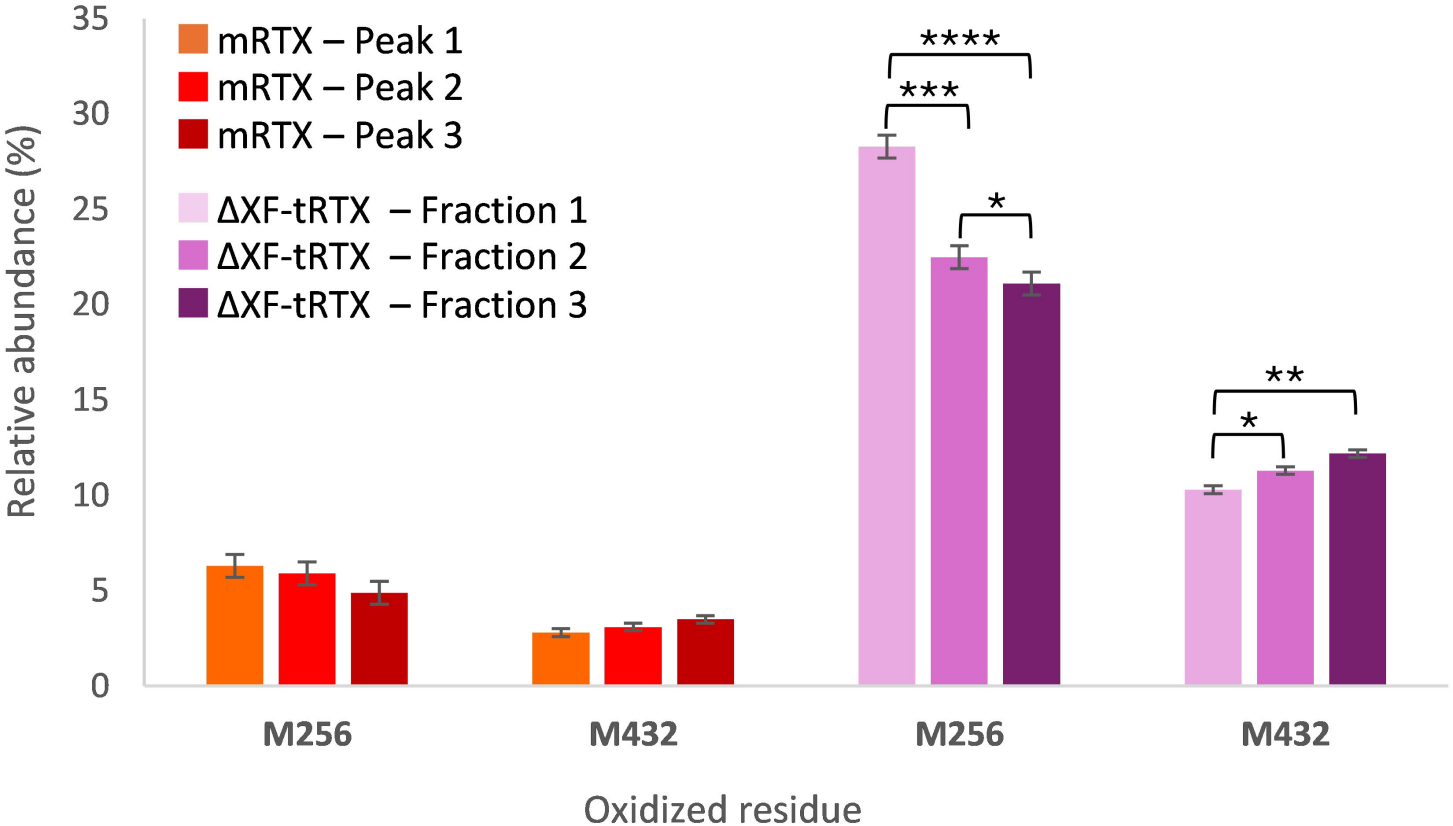
Percentage relative abundance of oxidized residues determined by peptide mapping analysis in fractions collected from affinity chromatography. The data are presented as mean values. SD was calculated by triplicate analysis. *p ≤ 0.05, **p ≤ 0.01, ***p ≤ 0.001, and ****p ≤ 0.0001 were calculated by Student’s *t*-test.

To investigate the abundance of PTMs other than glycosylation in the tobacco-derived plantibodies in comparison to m-RTX and to study their effect on the binding to FcγRIIIa receptor, peptide mapping analysis data were first considered. Significant differences were observed in the occurrence and abundance of PTMs, particularly oxidation. In **Table 3**, the detected oxidated peptides are listed together with their relative abundance in the different samples. To study if the detected PTMs might affect the binding to the Fc receptor and, consequently, the ADCC, different fractions of the eluting profile of m-RTX and ΔXF-tRTX samples in affinity chromatography were collected and analyzed by peptide mapping LC-HRMS. The distribution of modified peptides among the different fractions was established, looking for correlation between retention time in affinity chromatography and degree of protein modification. For m-RTX, three fractions were collected, corresponding to the three main peaks observed (see **Table 1**; **Figure 4**). Conversely, as ΔXF-tRTX displayed a single peak in affinity chromatography, three fractions were collected from the front, middle, and end of the peak. Once collected, each fraction was buffer-exchanged to allow protein denaturation and reduction/alkylation and then digested using trypsin. The resulting peptide mixtures were analyzed by RPLC-HRMS. Oxidation was found to be significantly higher in plantibodies compared to m-RTX, which is in agreement with the previous results (**Table 3**). Considering the major oxidation sites (Met256 and Met432), no significant differences were observed in the oxidation degree among the three major peaks of m-RTX (**Figure 5**). Differently, in the case of ΔXF-tRTX, a statistically significant different distribution was observed in the oxidation degree of Met256, which was found most abundant in the first eluting fraction of the peak. An opposite trend was observed, even with less statistical significance, for oxidated Met432, whose abundance in ΔXF-tRTX fractions increases with the retention time.

**Table 3:** PTMs detected in the mRTX and tRTX samples and their relative abundance.

Peptide	Chain	Site	Modification	Relative abundance (%)			
				m-RTX	Wt-tRTX	K-tRTX	ΔXF-tRTX
ASGYTFTSYNMHWVK	HC	M34	Oxidation	1.2	1.2	1.5	1.4
DTLMISR	HC	M256	Oxidation	3.8	11.1	17.0	21.7
WQQGNVFSCSVMHEALHNHYTQK	HC	M432	Oxidation	1.7	8.9	13.3	11.4
SSSTAYMQLSSLTSEDSAVYCAR	HC	M81	Oxidation	0.4	1.0	0.8	0.5
QIVLSQSPAILSASPGEKVTMTCR	LC	M21	Oxidation	0.4	0.4	0.4	0.7

## 3 Discussion

The production of mAbs in plants remains a promising answer to the very large-scale production demand that is expected to characterize the following years. Promoting a deeper understanding of the challenges associated with plant-based production of mAbs is essential and will prove to be highly valuable for future advancements. In this work, considering the importance of antibody effector functions, it has been shown how a deep characterization of attributes affecting FcγRIIIa binding can be useful to rationalize the production process in terms of glycosylation and also of other PTMs.

The anticancer drug RTX was successfully produced in engineered ΔXF *N. benthamiana* plants by using the vacuum agroinfiltration approach. Moreover, a glyco-optimized mAb was obtained without the need of using genetically engineered plants by simply adding the kifunensine mannosidase I inhibitor during the agroinfiltration process. We set up protocols for the rapid production of RTX that allowed us to obtain the final purified antibody in just 8 days after the agroinfiltration process. The expression yield in the plant extract (~100 mg/kg fresh weight) was sensibly lower compared to a previous study in which the expression levels ranged from 288 to 385 mg/kg (Kommineni et al., 2019). This is because plant viral-based expression vectors have a higher efficiency compared to the 35S T-DNA-based cassette used in our study. We obtained yields of the final highly purified antibody that were in the range of 50–75 mg/kg. The final product was highly homogeneous with minor degradation or aggregation products. Degradation of recombinant proteins produced in plants is a common phenomenon (Jutras et al., 2020). In particular, human mAbs produced in *N. benthamiana* through agroinfiltration technology can be subject to extensive proteolytic degradation by specific classes of plant proteases especially in the hinge region, leading to more than 50% degradation (Donini et al., 2015; Hehle et al., 2015).

The structural characterization suggested that the plantibodies have the same amino acid sequence as m-RTX and enabled the attribution of the degradation issue to the Fd portion. An identity characterization at semi-intact (reduced IdeS-generated fragment) level for wt-tRTX and rRTX produced in transgenic rice was already previously reported (Tengattini et al., 2022). Both antibodies showed the Fd-truncated species pQ1-T227 generated by the loss of C-terminal 13 amino acids. To our knowledge, in literature, there are no other examples of identity characterization at semi-intact level performed on glycovariants of plant-produced RTX. In the present work, we show that the Fd-truncated species pQ1-T227 is present in the wt-tRTX and K-tRTX but not in the ΔXF-tRTX. One could speculate that the lower proteolytic susceptibility observed for ΔXF-tRTX could be due to its higher glycosylation occupancy compared to wt-tRTX and K-tRTX. Indeed it is known from literature that deglycosylated mAbs have a higher susceptibility to proteolytic cleavage (Zheng et al., 2011; Liu et al., 2023). Most interestingly, the rice-produced rRTX also showed low glycan occupancy and the presence of the Fd-truncated species pQ1-T227 (Tengattini et al., 2022).

HILIC-MS analysis additionally allows, in a single analysis, the determination of glycoforms present in each sample (together with their relative abundance) and the estimation of glycosylation degree. The estimation of glycosylation degree is a crucial aspect in mAb characterization, especially when a new production system is explored, since aglycosylated antibodies lack effector functions and their half-life and serum stability are dramatically reduced (Zheng et al., 2011).

As expected, the wt-tRTX presented plant-specific glycans, whose presence has been regarded as a potential risk for immunogenic side effects, thus representing non-desired species (Bardor et al., 2003) even if there is no agreement in the literature on this topic, and clinical data show no apparent risk related to anti-plant glycan antibodies (Rup et al., 2017). The plantibody expressed in the presence of kifunensine, according to the literature (Kommineni et al., 2019), consists of a highly mannosilated species, mainly carrying a Man9 chain, with the absence of plant-specific glycans. Glycoengineered ΔXF plants produced a highly homogeneous glycosylation profile, consisting in a single, afucosylated glycoform carrying a G0 chain. All of the plantibodies showed incomplete glycosylation, although in the ΔXF-tRTX sample a high (mean: 91%, *n* = 5) and consistent (SD: 1.8%, *n* = 5) glycosylation degree was achieved. Similar glycosylation occupancy levels (~60%) for wt-tRTX were previously observed by us (Tengattini et al., 2022). It is known from literature that the production of recombinant IgG1 antibodies in *Nicotiana benthamiana* generally results in significant underglycosylation at the conserved Asn297 site (Göritzer et al., 2025). Indeed transiently expressed monoclonal IgG1 trastuzumab in the RNAi downregulated line *Nicotiana benthamiana* ΔXT/FT line had 60% to 70% occupancy levels (Göritzer et al., 2025). Using the same FX-KO line described in our study, Jansing and colleagues produced, by agroinfiltration, the human monoclonal antibody 2G12, obtaining occupancy levels of about 85% comparable to the ~90% obtained for ΔXF-tRTX (Jansing et al., 2019). It can be therefore hypothesized that occupancy levels could be influenced by the plant line.

Affinity chromatography, by using a FcγRIIIa column, was used to correlate the observed glycosylation profiles with receptor binding. According to the literature (Woodall et al., 2022), m-RTX showed three main peaks and galactosylation was shown to increase receptor affinity (Zhang et al., 2020). The wt-tRTX showed two main glycoforms, one carrying G0 and the other carrying G0F*X. Interestingly, only the G0 glycoform was retained by the affinity column, while the plant-specific glycans, Xyl and α1,3-Fuc, were shown to completely affect the binding to the receptor so that this glycoform eluted with the solvent front. This result highlights the detrimental effect of the presence of residual plant-specific glycosylation on ADCC, in accordance with previously reported studies. For example, Stelter et al. (2020) demonstrated, using SPR-based assays, that a plant-produced HIV antibody with typical plant glycosylation had a very limited capacity to engage the FcγRIIIa receptor.

G0 was also the only glycoform produced in ΔXF-glycoengineered plants. Lack of core fucose is known to greatly increase the affinity for FcγRIIIa receptor, which is due to a structural change enabling an additional favorable carbohydrate–carbohydrate interaction between the mAb Fc and glycans on FcγRIIIa, along with an additional hydrogen bond involving Tyr296 and FcγRIIIa (Ferrara et al., 2011; Woodall et al., 2022). Although an increased retention in affinity chromatography can be noticed for the afucosylated species comparing the elution time of G0 and G0F, this time shift does not reflect the improvement (up to 50-fold) in binding affinity and ADCC that has been established by SPR and biological assays. It must be pointed out that the immobilized receptor on the affinity column lacks Asn162 glycosylation and the removal of the N-glycan on FcγRIIIa reduces its affinity for afucosylated mAbs due to the abolition of carbohydrate–carbohydrate interactions (Cambay et al., 2019; Woodall et al., 2022). It can be speculated that the additional interaction involving the Tyr296 residue is responsible for the retention time shift that can been still observed in affinity chromatography.

The expression of RTX in the presence of kifunensine mannosidase I inhibitor successfully resulted in the production of a highly mannosylated RTX. Once analyzed by affinity chromatography, one peak was observed with retention time between the low- and medium-affinity peaks of m-RTX. No data or direct evidence in the literature supports the immunogenicity of high mannose glycan structures (Pacis et al., 2011), and several studies report enhanced FcγRIIIa binding and ADCC in antibodies with oligomannose-type glycans (Yu et al., 2012; Reusch and Tejada, 2015; Kommineni et al., 2019). These findings indicate glycoengineered high mannose mAbs as potential biobetters. However, core fucose is absent in the highly mannosylated glycoforms. Therefore, it remains unclear if the observed increase in FcγRIIIa binding and ADCC activity lies in the presence of multiple mannose moieties or in the absence of core fucose. Yu et al. showed that the mannosylated glycostructures could induce ADCC via FcγRIIIa binding but to a lesser extent than the hybrid and complex glycan structures (Yu et al., 2012). Our results agree with this evidence, showing that, in the comparison between the two afucosylated mAbs, G0 glycoform has a higher interaction with FcγRIIIa receptor compared to high mannose glycoforms.

Apart from glycans, other PTMs have shown the potential to affect mAb ADCC. Plantibodies have demonstrated to be particularly prone to oxidation. All of the samples showed a significant increase in oxidation, with Met256 and Met432 as the most susceptible sites. When the effect of PTMs on retention time in affinity chromatography was investigated, Met256 oxidation was found to have a higher abundance in the first eluting, less affine, fraction of ΔXF-tRTX peak. The same trend was not observed considering the three fractions collected for m-RTX, corresponding to the three major peaks. Woodall et al. used a similar approach to investigate attributes affecting the binding of RTX to FcγRIIIa V158 receptor. They collected the major peaks of RTX in affinity chromatography and, looking at PTMs, found out that no oxidation event affected retention and thus binding (Woodall et al., 2022). Our results confirmed their findings, suggesting that, in the case of a heterogeneously glycosylated mAb, glycans are the main factor driving affinity. Indeed when considering a single glycoform mAb, such as ΔXF-tRTX, in which the glycan influence can be regarded as minimal, only a marginal impact of Met256 oxidation can be observed. Differently, Met432 oxidation was not found to negatively affect the affinity to FcγRIIIa receptor. It must be highlighted that retention shift among non-oxidated and oxidated species suggests a minimal effect of Met256 oxidation on receptor binding and that this data alone does not enable to draw conclusions about the biological consequences of this modification on the activity of plantibodies. Further investigation is certainly required to clarify the significance of this influence which, based on the existing literature, might not be biologically relevant. In conclusion, this work reports the successful production of mAb RTX glycovariants in *N. benthamiana* following two different strategies and illustrates the potential—as well as the limitations—of using FcγRIIIa affinity chromatography to correlate structural features with receptor binding. While affinity chromatography has undeniable limitations (such as its inability to assess the effect of afucosylation on binding), it has also proven to be a powerful approach to study plantibody CQAs affecting the interaction with FcR.

As glycoengineering of mAbs becomes increasingly widespread and plant-based production remains a promising solution to meet the growing large-scale demand for mAbs, gaining a comprehensive understanding of plant-derived quality attributes (not only glycosylation) that affect effector function has a crucial role in guiding the development of new, effective plant-derived biosimilars or biobetters.

## 4 Materials and methods

### 4.1 Plant expression constructs

The rituximab heavy chain (HC) and light chain (LC) encoding genes were synthetically constructed by GenScript based on amino acid sequences from Protein Data Bank (PDB entry 4kaq). At the N-terminus of both genes, the secretory signal sequence PR1 UniProtKB - P08299 (PR1A_TOBAC) was fused. The synthetic sequences (hc and lc) were excised from pUC57 (Genscript) plasmid by digestion with BamHI and XmaI restriction enzymes and inserted into similarly digested pGEM-NOS plasmid (Lombardi et al., 2009) to be then transferred together with the nopaline synthase terminator (NOSter) into the binary vector pBI-Ω (Marusic et al., 2007) using BamHI/EcoRI restriction sites, yielding plasmids pBI-Ω-HC and pBI-Ω-LC. In this vector, gene expression is under the control of the constitutive Cauliflower Mosaic Virus 35S promoter (35SCaMV), the Ω translational enhancer sequence from Tobacco Mosaic virus, and the NOSter sequence. The pBI-Ω-p19 bearing the p19 silencing suppressor gene from AMCV was also used (Marusic et al., 2007). All synthetic genes were codon-optimized for expression in *N. benthamiana* using the OptimumGeneTM algorithm (GenScript).

### 4.2 Plant growth and transient expression in *N. benthamiana* plants

For plant growth, an ebb-and-flow-type irrigation with two floods in 24 h was used. Ionic Hydro Grow type nutrient solution (Growth Technology Ltd), 3 mL/L for germination and 5 mL/L for growth, was used together with Silicon (Growth Technology Ltd, 90 mL per liter). Germination was performed in rock wool blocks (Grodan 25/150) at a temperature of 24°C and with light at an intensity of 80 mmol m^2^/s- and 80% relative humidity. After 10 days from sowing, the seedlings were then transferred to the hydroponic growth module in Cultilene 7.5 × 7.5-cm culture substrate (Grodan) with a photoperiod of 16-h light and 8-h dark at a density of 36 plants per square meter at a temperature of 24°C and 60% relative humidity. Different light recipes using commercially available LEDs (Valoya AP673L, NS12, and G2) were studied in order to assess plant growth with the same light intensity of 100 μmol m^2^/s^-1^. For each lighting condition, measurements of plant height were performed on days 30, 35, 40, and 43 after sowing, and leaf fresh weight was measured 43 days post-sowing.

Transient expression in plant leaves was performed by vacuum agroinfiltration. Three *A. tumefaciens* (LBA 4404, Thermo Fisher Scientific, Rockford, IL, USA) strains harboring the pBI-Ω-HC and pBI-Ω-LC and pBI-Ω-p19 plant expression vectors were grown separately in 500 mL of LB medium overnight at 28°C on an orbital shaker at 250 rpm. Bacteria cultures were separately pelleted/sedimented by centrifugation at 4,000 × *g* and resuspended in infiltration buffer (10 mM 2-(N-morpholino) ethanesulfonic acid (MES, Millipore, 106126) and 10 mM magnesium chloride hexahydrate (Sigma-Aldrich, 255777), pH 5.8). To obtain glyco-modified antibodies, the kifunensine mannosidase I inhibitor (Sigma-Aldrich, K1140) was added at this step in the infiltration buffer at a final concentration of 0.5 µM. *Agrobacterium* suspensions harboring the different vectors were mixed (pBI-Ω−HC, pBI-Ω-LC, and pBI-Ω-p19) to reach the final optical density (OD600) of 0.4 for each strain. Agroinfiltration was performed either using DXF *N. benthamiana* plants (at the six- to seven-leaf stage, about 35 days post-sowing) or wild-type *N. benthamiana* plants when using the kifunensine mannosidase I inhibitor. Infiltration was performed by completely submerging each plant in the *Agrobacterium*-containing solution inside a vacuum chamber. Vacuum was then applied, reaching ~10 mmHg, and then quickly released. Infiltration was confirmed visually by observing the infiltrated areas as translucent. The plants were then placed in the greenhouse, and leaf sampling was performed at set time points (1 to 6 d.p.i). Leaves of the same age (from the “middle leaf” position, typically leaf 4 and 5 from the bottom) from three individual plants were collected and stored in liquid N_2_. For antibody purification, batches of 40 g of agroinfiltrated leaves of all ages were typically collected at 6 d.p.i., frozen in liquid N_2_, and stored at -80°C.

### 4.3 Western blot analysis, ELISA, and Coomassie staining

Protein extracts for the analysis of agroinfiltrated leaves were obtained by grinding 0.2 mg of tissue in liquid nitrogen and by homogenization with pestle in PBS, pH 7.5, supplemented with a protease inhibitor cocktail (cOmpleteTM; Roche, 111836153001) in a 1:2 weight/volume ratio. The extracts were centrifuged at 20,000 × *g* for 20 min, and the supernatants were recovered. For quantification of HC and LC expression, agroinfiltrated leaves of the same age were analyzed by Western blot. Separation was performed on SDS-12% PAGE acrylamide gel, and proteins were electrotransferred to a PVDF membrane (Millipore, IPVH0001) using a Semi-Dry Transfer Unit (Hoefer TE70, GE Healthcare, 80-6210-34). Membranes were blocked with PBS containing 4% skimmed milk (w/v) overnight at 4°C before adding anti-human γ chain HRP-conjugated produced in goat using purified human IgG as the immunogen (Sigma-Aldrich, A8419 lot 052M4808), at a dilution of 1:5,000, in PBS containing 2% skimmed milk (w/v, Millipore 70166). Proteins were detected by enhanced chemiluminescence (ECL, Prime Western blotting detection reagents, RPN2232). To quantify mAb expression, crude leaf extracts from agroinfiltrated leaves under different lighting conditions were analyzed by Double-Antibody-Sandwich (DAS)-ELISA. Leaf tissue (100 mg) was ground in liquid N_2_ in 500 μL of phosphate-buffered saline, pH 7.2 (PBS), containing protease inhibitors (CompleteTM, Roche, 111836153001). The extract was cleared by centrifugation at 20,000 × *g*, 4°C, for 20 min, and the supernatant was quantified for total soluble proteins (TSP) using the Bradford colorimetric assay (Bio-Rad Protein Assay Dye Reagent Concentrate, 5000006). The captured antibody (anti-human γ chain Clone GG-4 Mouse Ascites Fluid) was coated directly onto Nunc-Immuno Maxisorp wells at a concentration of 2 μg/mL in PBS. Purified human IgG was used as the immunogen (Sigma-Aldrich, I6010). Plates were blocked with 4% (w/v) milk in PBS at 37°C for 2 h, and different dilutions of the leaf extracts, normalized for TSP concentration, were added to the wells (100 μL) and incubated for 2 h at 37°C. The anti-human κ chain HRP-conjugated antibody from goat obtained using purified Human Kappa Light Chain as immunogen (Thermo Fischer Scientific, A18853) at a dilution of 1:5,000 in PBS containing 2% (w/v) milk was incubated at 37°C for 1 h. Enzymatic activity was at 405 nm using a microtiter plate reader (TECAN-Sunrise, 11831) using 2,2-azino-di-3-ethylbenz-thiazoline sulphonate (ABTS, Seracare Life Sciences, 5120-0032). As positive control, the commercial RTX (m-RTX; MabThera^®^ produced in Chinese hamster ovary (CHO) cells, manufactured by Roche as 10 mg/mL solution in formulation buffer, pH 6.5) was used at different dilutions (ranging from 1 to 100 ng). To assess the expression and assembly of the antibodies, plant extracts were separated by non-reducing SDS-10% PAGE acrylamide gel, and transferred proteins were detected as described above. Protein-A purified plant antibodies were separated by reducing or non-reducing SDS-10% PAGE, and proteins were visualized by Coomassie blue staining.

### 4.4 Antibody purification by protein-A affinity chromatography and SEC analysis

The antibodies were purified from agroinfiltrated *N. benthamiana* plants by protein A affinity chromatography. Batches of leaves (40 g) were ground in liquid nitrogen in the presence of 4% w/w PVPP and mixed with 80 mL of PBS, 0.2% Tween, and 1 mM phenylmethanesulfonyl fluoride (Sigma-Aldrich, PMSF 78830) and homogenized using an Ultra-Turrax homogenizer T25 (IKA, 0003725000). After Miracloth (Sigma-Aldrich, 475855) paper filtration and centrifugation at 20,000 × *g* for 20 min, the supernatant was then adjusted with NaOH to pH 7.5, incubated for 30 min at 4°C, and then centrifuged at 20,000 × *g* for 20 min. Extracts were then filtered through a 0.45-µM PES membrane (Millipore, SLHPR33RS) before purification. The resulting supernatant was loaded onto a protein A affinity column (1 mL HiTrap™ Protein A FF, Cytiva, 17508001), previously equilibrated with PBS, at a flow rate of 1 mL/min. The column was washed with 10 mL of PBS (10 column volumes), and the antibody was eluted with 100 mM citric acid, pH 3.0, and buffered with one-tenth volume of 3 M Tris-HCl, pH 11.0. The eluted fractions were analyzed by SDS-PAGE, followed by Coomassie staining. Antibody-containing fractions were pooled and passed through a PD10 column (Cytiva, GE17-0851-01) in PBS according to manufacturer instructions. Antibody concentration was determined spectrophotometrically by absorbance at 280 nm. The purified antibodies were analyzed by size exclusion chromatography on a calibrated SuperdexTM 200 5/150 GL column (GE Healthcare, GE28-9909-45) or SuperdexTM 75 10/300 GL column (GE Healthcare, GE17-5174-01) eluted in PBS at a flow rate of 0.3 or 0.7 mL/min, respectively, using an ÄKTA FPLC P920 instrument (GE Healthcare, 256055416326) thermostated at 20°C, essentially as described before (Lombardi et al., 2010). Protein absorbance expressed as absorption units (mAU) was measured at 280 nm. Column calibration was performed using gel filtration calibration kits (Low 28403842 and High 28403841 Molecular Weight GE Healthcare), according to the manufacturer’s instructions. As a control, commercial m-RTX was used (**Supplementary Figure S3**).

### 4.5 Antibody structural characterization

All of the reagents used for characterization were of analytical grade. Water was obtained from a Milli-Q Integral purification system from Merck Millipore. Organic solvents were purchased from Sigma-Aldrich, Merck.

#### 4.5.1 Peptide mapping analysis

The preparation of reagents and solutions was performed in accordance with Tengattini et al. (2022). Briefly, disulfide bridge reduction was carried out on 100 µL of 0.5 mg/mL mAb solutions in 6 M guanidine hydrochloride (Sigma-Aldrich, Merck, G3272) and 1 mM EDTA (Sigma-Aldrich, Merck, EDS) in 0.25 M tris buffer (Sigma-Aldrich, Merck, T1503), pH 7.5, mixed with 10 µL of 0.5 M dithiothreitol (DTT; Sigma-Aldrich, Merck, 43819) in water. The solutions were incubated at 37°C for 30 min. Alkylation was performed by adding 24 µL of 0.5 M iodoacetamide (IAA; Sigma-Aldrich, Merck, I6125) in water at room temperature for an additional 30 min in the dark. Finally, 10 µL of 0.5 M DTT was added to quench the residual IAA. Reduced and alkylated samples were loaded on a PD-10 Sephadex G-25 desalting column (GE Healthcare Bio-Sciences AB) and eluted with the digestion buffer. Fractions of 700 µL each were collected and measured at 280 nm. The fraction having a higher absorbance was freeze-dried and subsequently solubilized in 250 µL of water. Digestion was carried out on each sample by mixing 90 μL of reconstituted fraction and 3.6 μL of 1 mg/mL trypsin in 0.05 M acetic acid (Promega, V5117) and incubating the solutions at room temperature under continuous stirring. After 20 h, 1.8 μL of TFA (ROMIL Ltd, HA853S) was added to stop the digestion, and the mixture was stored at 4°C. LC-MS separation was carried out with a Symmetry 300 C18 column (1.0 × 150 mm, 3.5 μm, 300 Å; Waters, 186000185) on an ExionLC system (SCIEX) equipped with mobile-phase online degasser, quaternary pump, autosampler, and column thermostated compartment. MS detection was carried out by using a X500 QTOF mass spectrometer (SCIEX) with an ESI source. The LC-MS system was controlled by using SCIEX OS software (1.7 version). Mobile phases consisting of 0.1% TFA (v/v) in water (A) and 0.1% TFA (v/v) in acetonitrile (B) were used with a 115-min linear gradient (from 2% B to 45% B) at a flow rate of 0.075 mL/min. Column temperature and injection volume were fixed at 60°C and 10 μL, respectively. UV detection was performed at 215 nm. The following MS parameters were applied: positive mode, curtain gas 25 psi, ion source gas 1 35 psi, ion source gas 2 45 psi, temperature 300°C, polarity positive, ion spray voltage 5,500 V, CAD gas 7, time bins to sum 6, TOF start mass 400 Da, TOF stop mass 2,500 Da, accumulation time 0.1 s, declustering potential 80 V, and collision energy 10 V. Data processing was performed using Explorer for SCIEX OS software (SCIEX) by comparing experimental data with RTX amino acidic sequence in FASTA. Peptides were selected by using approximate LC peak width 20 s, minimum intensity in counts 3, chemical noise intensity multiplier 1.5, mass tolerance 10 ppm, maximum charge 4, and maximum missed cleavages 5.

#### 4.5.2 Reverse-phase LC-HRMS analysis at subunit level

Each mAb was treated with IdeS protease (1.5 unit of IdeS Protease per 1 μg of IgG; Promega, V7511) in the digestion buffer, consisting of 0.05 M sodium phosphate (Sigma-Aldrich, Merck, 71505) and 0.15 M sodium chloride (ITW Reagents, 131659), pH 6.6, and incubated overnight at 37°C. Reduction of the digested mAbs was performed by using 0.02 M DTT and incubating the mixtures at 37°C for 30 min. RP analyses were performed on an ExionLC system (SCIEX) equipped with mobile-phase online degasser, quaternary pump, autosampler, and column thermostated compartment and coupled to an X500 QTOF high resolution-mass spectrometer (SCIEX) with an ESI source. The system was controlled by using SCIEX OS software (1.7 version). Separation of mAb subunits was performed using an AdvanceBio RP-mAb C4 column (4.6 ×50 mm, 3.5 µm, 450 Å; Agilent Technologies, 799775-904) and a mobile phase composed of 0.1% (v/v) formic acid (FA; ROMIL Ltd, HA353S) in water (A) and 0.1% (v/v) FA in ACN (B). The elution program consisted in a linear gradient from 20% to 60% B in 10 min. Flow rate, column temperature, and injection volume were set at 1 mL/min, 80°C, and 5 μL, respectively. Full-scan MS experiments were carried out using the following instrumental conditions: curtain gas 40 psi, ion source gas 1 45 psi, ion source gas 2 55 psi, temperature 450°C, polarity positive, ion spray voltage 5,500 V, CAD gas 7, time bins to sum 6, TOF start mass 600 Da, TOF stop mass 3,000 Da, accumulation time 1 s, declustering potential 150 V, and collision energy 10 V.

#### 4.5.3 Glycoprofiling by HILIC-UV-MS analysis at subunit level

Reduced IdeS digests were analyzed using an AdvanceBio Glycan Mapping column (2.1 × 150 mm, 1.8 μm, 300 Å; Agilent Technologies, 859700-913). Mobile phases were composed of acetonitrile (A) and water (B), both containing 0.1% v/v TFA. Chromatographic conditions consisted of a linear gradient from 25% to 40% B in 20 min, flow rate of 0.25 mL/min, 50°C, and injection volume of 5 μL. Full-scan MS experiments were performed by applying the following parameters: curtain gas 35 psi, ion source gas 1 45 psi, ion source gas 2 55 psi, temperature 400°C, polarity positive, ion spray voltage 5,500 V, CAD gas 7, time bins to sum 6, TOF start mass 1,300 Da, TOF stop mass 3,000 Da, accumulation time 1 s, declustering potential 200 V, and collision energy 10 V.

#### 4.5.4 Glycopeptide mapping analysis

Chromatographic HILIC separation of tryptic digests were performed on a Dionex UltiMate 3000 HPLC system (Thermo Scientific) equipped with mobile-phase online degasser, quaternary pump, autosampler, column thermostated compartment, and variable wavelength detector. MS detector as a linear ion trap mass spectrometer (LTQ) was equipped with electrospray ion source (ESI) (Thermo Scientific). The HILIC method entailed the use of an AdvanceBio Glycan Mapping column (2.1 × 150 mm, 2.7 μm, Agilent Technologies, 68775-913), and mobile phases were composed of acetonitrile/10 mM ammonium formate, pH 4.0, 90/10 (v/v) (A) and 10 mM ammonium formate, pH 4.0 (B). After an isocratic step of 2 min at 0% B, glycopeptides were eluted using a linear gradient from 0% to 45% B in 60 min. Flow rate, temperature, and injection volume were set at 0.3 mL/min, 50°C, and 20 μL, respectively. The following MS parameters were applied: positive ion mode, source voltage 4.5 kV, capillary voltage 31 V, sheath gas flow rate 40 (arbitrary units), auxiliary gas flow rate 10 (arbitrary units), capillary temperature 250°C, tube lens voltage 95 V, scan range 400–2,000 m/z in full-scan mode, and collision-induced dissociation (CID) with normalized collision energy of 35.0 for MS/MS spectra.

### 4.6 Affinity chromatography with FcγRIIIA column

#### 4.6.1 Plantibody analysis

Analyses were performed using a HPLC 1200 Series (Agilent Technology). A TSKgel FcR-IIIA-NPR column (4.6 × 75 mm, 5 µm; Tosho Bioscience, 0023513) was used. Separations were carried out under pH gradient conditions using as mobile phase 0.05 M sodium citrate tribasic dihydrate (Sigma-Aldrich, Merck, 6132043), 0.150 M sodium chloride, pH 6.5 (A), and 0.05 M sodium citrate tribasic dihydrate, 0.150 M sodium chloride, pH 4.5 (B). After an isocratic step of 10 min at 0% B, elution was achieved by a linear gradient from 0% to 100% of B in 40 min. The flow rate was set at 0.375 mL/min, and the temperature was at 20°C. The injection volume was 20 µL, and the UV absorbance was monitored at 280 nm.

#### 4.6.2 FcγRIIIA column fraction collection and analysis

Peak collection was manually performed after UV detection. The collected solutions were submitted to ultracentrifugation using Amicon Ultra- 0.5-mL centrifugal filters with NMWL of 10 kDa (Merk Millipore, 41104916) and an Eppendorf 58024R centrifuge. Samples were submitted to two centrifugation cycles at 13,000 × *g* at 4°C for 10 min, collected in 1 mM sodium phosphate, 3 mM sodium chloride, pH 6.6, and finally dried using Concentrator plus (Eppendorf) at 45°C for 2 h. IdeS digestion was performed by reconstituting the samples with 10 µL of 50 mM sodium phosphate and 150 mM sodium chloride, pH 6.6, and adding 2 µL of IdeS solution (5 mg/mL). The digestion was carried overnight at 37°C. Reduction was performed by adding 2 µL 0.1 M DTT in 0.2 M sodium phosphate and incubating the reaction at 37°C under stirring for 30 min. The processed samples were then analyzed in HILIC-MS for glycoform assignments using the method reported in Section 4.5.3.

#### 4.6.3 FcγRIIIA column fraction collection and peptide mapping analysis

Fractions collected from affinity chromatography, corresponding to the three major peaks of m-RTX and to the start, middle, and ending parts of the ΔXF-tRTX peak, were processed for tryptic digestion. First, the samples underwent ultrafiltration using Amicon centrifugal filters with NMWL of 10kDa to exchange the buffer with 6 M guanidine hydrochloride, 1 mM EDTA, and 100 mM Tris-HCl, pH 7.8. Reduction was then carried out by adding DTT from a 0.5-M solution to a final concentration of 5 mM, followed by incubation at 37°C for 30 min. After reduction, the samples were alkylated by adding IAA from a 0.5-M solution to a final concentration of 10 mM. The reaction was performed in the dark at room temperature for 30 min. To quench the excess alkylating agent, an additional aliquot of DTT from the 0.5-M solution was added to achieve a final concentration of 5 mM. To remove the denaturing buffer, the samples were subjected to a second ultrafiltration step using Amicon filters and transferred to 1 M urea (Sigma-Aldrich, Merck, U0631) and 50 mM Tris-HCl at pH 7.8. Finally, trypsin was added at a 1:20 enzyme-to-substrate ratio (w/w), and digestion was carried out for 4 h at 37°C. The reaction was then stopped by adding formic acid to a final concentration of 2.5% (v/v). RP-LC-HRMS analyses were carried out on an Ultimate 3000 LC UHPLC system (Thermo Scientific) coupled to a Q Exactive™ Orbitrap with a heated electrospray ionization (HESI) source. A Kinetex EVO C18 column (100 × 2.1 mm, 5 µm, 100 Å; Phenomenex, 00D-4633-AN) was used, and the mobile phase was composed of water (A) and ACN (B), both containing 0.1% (v/v) formic acid. The elution was performed by linearly increasing from 2% to 45% B in 45 min. Flow rate, temperature, and injection volume were set at 0.3 mL/min, 60°C, and 20 µL, respectively. Acquisition was performed in positive ion mode. HRMS parameters were set as follows: spray voltage, 3.7 kV; sheath gas flow, 30; auxiliary gas flow, 15; capillary temperature, 320°C; mass range, 350–1,800 m/z; AGC, 3 × 106; resolution, 7 × 104, and maximum injection time, 120 ms. MS/MS data were obtained by high-energy collisional dissociation (HCD) fragmentation and acquired in data-dependent mode with dynamic exclusion (repeat count of 3, exclusion duration of 10 s), AGC, 1 × 105; maximum injection time, 200 ms; isolation window, 2.0 m/z, and normalized collision energy, 28%. MaxQuant (v2.6.2.0) was used for data analysis, PTM identification, and label-free quantification. A maximum of two missed cleavages was allowed, and peptides were screened for variable modifications, including oxidation, dioxidation, deamidation, pyroglutamate formation, and acetylation.

### 4.7 Statistical analysis

Statistical analysis was performed using GraphPad Prism for Windows (GraphPad Software, La Jolla, CA, USA; www.graphpad.com). A two-tailed Student’s *t*-test Welch’s correction for unpaired samples was used. *P*-values <0.05 were considered statistically significant.

## Data Availability

The original contributions presented in the study are publicly available. This data can be found here: https://data.mendeley.com/datasets/d74sm33tfz/1.
