# Supplementary material for: Plant-related quality attributes affecting FcγRIIIa binding: affinity chromatography analysis of rituximab glycovariants from Nicotiana benthamiana
Source: Front Plant Sci. 2025 Jun 27;16:1607403. doi: 10.3389/fpls.2025.1607403 (PMC12246787; doi:10.3389/fpls.2025.1607403)
Supplement: Supplementary file 1 [file DataSheet1.docx]

Supplementary Material

**Supplementary Figure 1.** *N. benthamiana* plants growth with different light conditions. Different light recipes using commercially available LEDs (Valoya AP673L, NS12 and G2) were studied in order to assess plant growth with the same light intensity of 100 mmol m2 / s-1. (A) left panel plants grown in the hydroponic facility, right panel examples of plants grown with the different lights at day 40. For each lighting condition measurements of plant height were performed on day 30, 35, 40 and 43 after sowing (B) and leaf fresh weight was measured 43 days post-sowing (C). Values are the mean ± standard deviation (SD) (n=10). Unpaired two-tailed Student’s t-test was performed. (B) * p<0.05 for G2 vs AP673L and NS12 on day 35 and 40 and * p<0.05 between all lighting conditions at day 43. (C) * p<0.05 for G2 and AP673L vs NS12 on day 43.

**
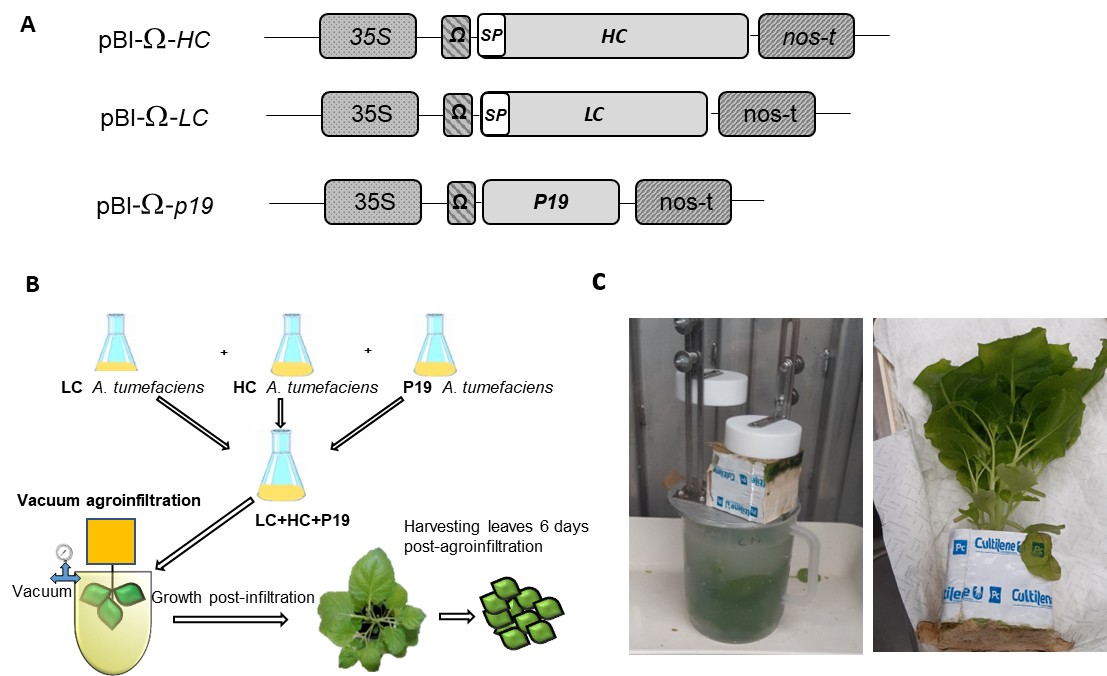
**

**Supplementary Figure 2.** Schematic representation of the HC, LC and the Artichoke Mottle Crinkled virus P19 silencing suppressor protein encoding sequences cloned in the pBI-Ω plant expression vector (A). In this vector, gene expression is under the control of the constitutive Cauliflower Mosaic Virus 35S promoter (35SCaMV), the Ω translational enhancer sequence from Tobacco Mosaic virus and the NOS-ter sequence (nos-t). (B) Schematic representation of the vacuum agroinfiltration process used for antibody expression in *N. benthamiana*. Plants were co-infiltrated using vacuum with an *Agrobacterium tumefaciens* solution containing a 1:1:1 mix of bacterial clones containing the HC the LC and the P19 silencing suppressor protein encoding genes. Example of an agroinfiltrated plant (C).

**
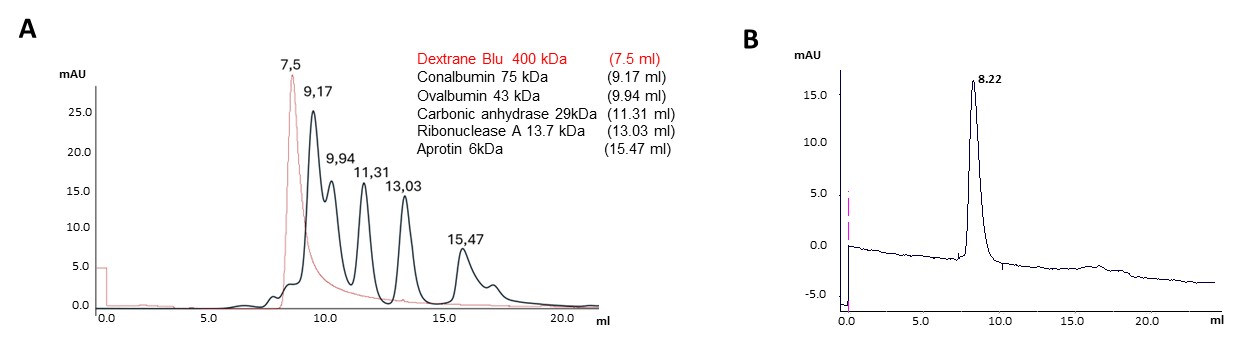
**

**Supplementary Figure 3.** Size-exclusion chromatography analysis on SuperdexTM 75 10/300 GL column. Samples were eluted in PBS at a flow rate of 0.7 mL/min, using an ÄKTA FPLC P920 instrument thermostated at 20 °C. Protein absorbance expressed as Absorption Units (mAU) was measured at 280 nm. Column calibration was performed using gel filtration calibration kits (A), according to manufacturer instructions. As a control, the commercial m-RTX was used (B).

**Supplementary Table 1.** Spectra characteristics of the commercial LED lights used in this study (Valoya AP673L, NS12). Photosynthetically active radiation (PAR); R:FR (red: far red ratio); B:G (blue: green ratio); B: R (blue: red).

|  | | **NS12** | **AP673L** | **G2** |
| --- | --- | --- | --- | --- |
| Blue | 400-500 nm | 20% | 10% | 9% |
| Green | 500-600 nm | 38% | 19% | 2% |
| Red | 600-700 nm | 36% | 63% | 66% |
| Far-Red | 700-800 nm | 6% | 8% | 23% |
| PAR | 400-700 nm | 94% | 91% | 77% |
| B:G Ratio |  | 0.5 | 0.6 | 3.6 |
| B:R Ratio |  | 0.5 | 0.16 | 0.13 |
| B:FR Ratio |  | 9.1 | 7.9 | 2.8 |

**Supplementary Table 2.** Data obtained from glycopeptide mapping analysis of mRTX and tRTX mAbs. GN= N-acetyl glucosamine; G= galactose; Man= mannose; F= α(1,6)fucose; F*=α(1,3)fucose; X= xylose.

| **Glycopeptide detected m/z** | **Glycan** | **Relative abundance (%)** | | | |
| --- | --- | --- | --- | --- | --- |
|  |  | **mRTX** | **wt-tRTX** | **Kif-tRTX** | **ΔXF-tRTX** |
| 1180.75 | 2 GN, 3 Man, F*, X |  | 3.1 |  |  |
| 1203.94 | Man5 | 1.8 |  |  |  |
| 1244.83 | G0 |  | 52.5 | 1.7 | 100 |
| 1282.33 | 3 GN, 3 Man, F*, X |  | 2.8 |  |  |
| 1317.92 | G0F | 46.1 |  |  |  |
| 1317.92 | G0F* |  | 2.3 |  |  |
| 1365.94 | Man7 |  |  | 5.0 |  |
| 1383.98 | G0F*X |  | 36.0 | 1.1 |  |
| 1398.99 | G1F | 43.7 |  |  |  |
| 1447.01 | Man8 |  | 1.9 | 12.9 |  |
| 1480.06 | G2F | 8.4 |  |  |  |
| 1528.08 | Man9 |  | 1.3 | 79.3 |  |
